# The roles of epigenetic modifications in the regulation of auxin biosynthesis

**DOI:** 10.3389/fpls.2022.959053

**Published:** 2022-08-09

**Authors:** Jun-Li Wang, Dong-Wei Di, Pan Luo, Li Zhang, Xiao-Feng Li, Guang-Qin Guo, Lei Wu

**Affiliations:** ^1^Ministry of Education (MOE) Key Laboratory of Cell Activities and Stress Adaptations, School of Life Sciences, Lanzhou University, Lanzhou, China; ^2^Gansu Province Key Laboratory of Gene Editing for Breeding, School of Life Sciences, Lanzhou University, Lanzhou, China; ^3^State Key Laboratory of Soil and Sustainable Agriculture, Institute of Soil Science, Chinese Academy of Sciences, Nanjing, China; ^4^College of Life Science and Technology, Gansu Agricultural University, Lanzhou, China; ^5^Basic Forestry and Proteomics Research Center, College of Life Sciences, Fujian Agriculture and Forestry University, Fuzhou, China

**Keywords:** auxin biosynthesis, epigenetic modifications, histone H3 methylation, histone acetylation, chromatin remodeling, histone H2B monoubiquitination, DNA methylation

## Abstract

Auxin is one of the most important plant growth regulators of plant morphogenesis and response to environmental stimuli. Although the biosynthesis pathway of auxin has been elucidated, the mechanisms regulating auxin biosynthesis remain poorly understood. The transcription of auxin biosynthetic genes is precisely regulated by complex signaling pathways. When the genes are expressed, epigenetic modifications guide *mRNA* synthesis and therefore determine protein production. Recent studies have shown that different epigenetic factors affect the transcription of auxin biosynthetic genes. In this review, we focus our attention on the molecular mechanisms through which epigenetic modifications regulate auxin biosynthesis.

## Introduction

As an important plant growth regulator, auxin plays a central role in regulating the growth and development of plants (Woodward and Bartel, 2005; Enders and Strader, 2015; Lavy and Estelle, 2016; Weijers et al., 2018; Semeradova et al., 2020). It is well-established that the key to auxin's control of growth and development is through its gradient distribution (Tanaka et al., 2006; Vieten et al., 2007). According to the classical view in the auxin field, auxin is produced in the shoot apical meristems, young leaves, and flower buds and is distributed over a gradient through Polar Auxin Transport (PAT) (Teale et al., 2006). More recently, local auxin biosynthesis has also been considered to play an important role in the formation of auxin gradients. Local auxin biosynthesis is regulated by diversified signaling pathways and guides plant growth and development and response to environmental stimuli (Brumos et al., 2018; Lv et al., 2019). The regulatory mechanisms underlying auxin biosynthesis include transcriptional regulation and posttranslational modifications (Casanova-Saez et al., 2021).

As one of the primary modes of transcriptional regulation, epigenetic modifications of the genome, which mainly include histone modifications in nucleosomes and DNA methylation modifications, change the structure of chromosomes or change the spatial structure of the modified DNA, resulting in gene silencing or overexpression. A number of reviews have summarized the functions of different epigenetic mechanisms in auxin signaling, transport, and metabolism (Yamamuro et al., 2016; Do et al., 2019; Mateo-Bonmati et al., 2019; Nguyen et al., 2020; Wojcikowska et al., 2020), two of which focused on epigenetic contributions to auxin biosynthesis. Mateo-Bonmati et al. (2019) comprehensively described the relationships between epigenetic modifications and auxin homeostasis. Do et al. (2019) highlighted the roles of epigenetic modifications in the *YUC* family genes, which are the most important genes in the auxin biosynthesis pathway. This review, based on emerging results, aims to provide an updated overview and some hypotheses about the effects of epigenetic modification on the regulation of auxin biosynthesis (Table 1).

**Table 1:** Epigenetically regulated auxin biosynthetic genes and the factors involved in the epigenetic modification.

**Auxin biosynthetic and metabolic genes**	**Factors responsible for epigenetic regulation**	**References**
*YUC10*	FIS-PRC2 complex	Figueiredo et al., 2015
*YUCs*	TFL2/LHP1	Rizzardi et al., 2011
*YUC1* and *YUC4*	SUP-LHP1-PRC2 complex	Xu et al., 2018
*YUCs*	JMJ12/REF6	Cui et al., 2016; Li et al., 2016; Yan et al., 2018
*YUC8*	JMJ14, JMJ15, and JMJ18	Cui et al., 2021
*YUC10*	EML1 and EML3	Milutinovic et al., 2019
*YUC8*	FCA	Lee et al., 2014
*YUC8*	PIF7-MRG2 complex	Bu et al., 2014; Xu et al., 2014; Peng et al., 2018
*GH3.3*	bZIP11-ADA2b-GCN5/HAG1 complex	Weiste and Droge-Laser, 2014
*YUC8*	SWR1 chromatin remodeling complex	Tasset et al., 2018; van der Woude et al., 2019
*YUC8*	HDA9-PWR complex	Tasset et al., 2018; van der Woude et al., 2019
*YUC8*	INO80 chromatin remodeling complex	Xue et al., 2021
*YUC4*	GCN5/HAG1	Poulios and Vlachonasios, 2018
*YUC9*	ARP6	Lee and Seo, 2017
*IAMT1* and *YUC6*	SWI3B	Han et al., 2018; Lin et al., 2021
*TSB1, WEI7/ASB1, YUC7*, and *AMI1*	HUB complex	Zhang et al., 2021
*YUC2*	*miRNA169, siRNA Locus_77297*, DRM1, and DRM2	Zhang et al., 2017; Gyula et al., 2018

## Main pathway for IAA biosynthesis

Biochemical and genetic evidence has shown that the major natural auxin, indole-3-acetic acid (IAA) is synthesized through two major pathways: the Tryptophan-Independent (TI) pathway and the Tryptophan-Dependent (TD) pathway (Woodward and Bartel, 2005; Normanly, 2010; Ljung, 2013; Casanova-Saez and Voss, 2019; Casanova-Saez et al., 2021). While the TD pathway is fairly well-understood, little is known about the TI pathway (Gomes and Scortecci, 2021).

**Figure 1:**
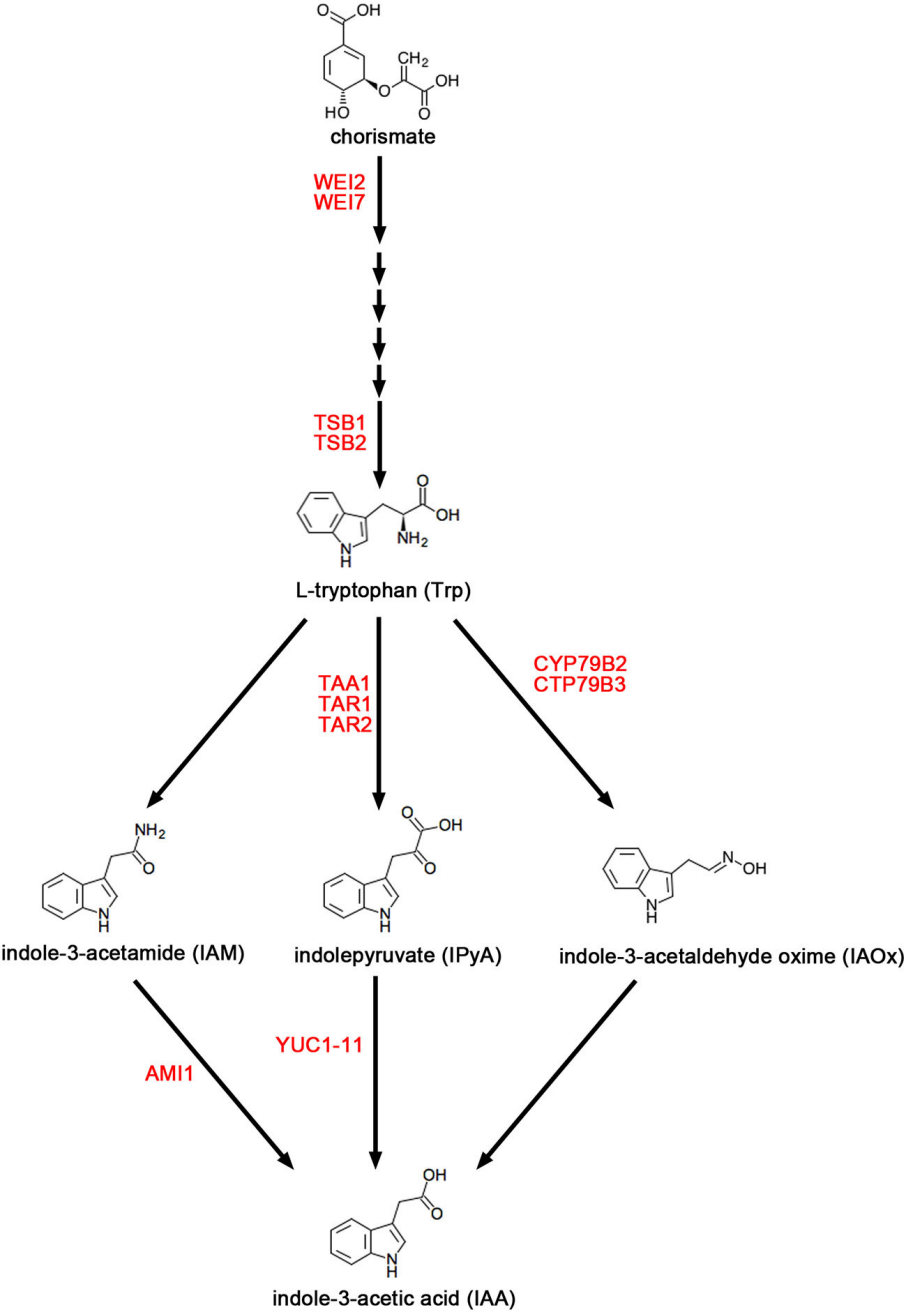
The main pathways of auxin biosynthesis. The figure shows three parallel Trp-dependent (TD) IAA biosynthesis pathways in *Arabidopsis*, namely the indole-3-acetamide (IAM) pathway, and the indole-3-pyruvic acid (IPyA) pathway, and the indole-3-acetaldoxime (IAOx) pathway. Enzymes mentioned in this review are in red font.

As shown in Figure 1, the Tryptophan (Trp) biosynthesis pathway includes six critical steps (Maeda and Dudareva, 2012). The rate-limiting step is catalyzed by anthranilate synthase, which contains two subunits, *WEAK ETHYLENE INSENSITIVE 2* (*WEI2*)*/ANTHRANILATE SYNTHASE ALPHA-SUBUNIT 1* (*ASA1*) and *WEI7/ANTHRANILATE SYNTHASE BETA-SUBUNIT 1* (*ASB1*) (Stepanova et al., 2005, 2008). Some publications reported that both *wei2* and *wei7* mutants exhibit auxin-deficient phenotypes triggered by reduced endogenous auxin (Stepanova et al., 2005; Di et al., 2016a; Veloccia et al., 2016). In the TD pathway, there are several parallel pathways downstream of Trp for IAA biosyntheses, such as the indole-3-pyruvic acid (IPyA) pathway, the indole-3-acetamide (IAM) pathway, and the indole-3-acetaldoxime (IAOx) pathway (Ljung, 2013; Di et al., 2016c; Casanova-Saez and Voss, 2019; Casanova-Saez et al., 2021). The IPyA pathway has been established as the main pathway of auxin biosynthesis (Mashiguchi et al., 2011; Won et al., 2011; Zhao, 2014) and consists of a two-step reaction. The TRYPTOPHAN AMINOTRANSFERASE OF *ARABIDOPSIS* (TAA) family proteins have overlapping functions and catalyze the conversion of Trp to IPyA (Stepanova et al., 2008; Tao et al., 2008; Yamada et al., 2009; Zhou et al., 2011). This family of proteins include three homologous proteins TAA1/WEAK ETHYLENE INSENSITIVE 8 (WEI8)/TRANSPORT INHIBITOR RESPONSE 2 (TIR2)/SHADE AVOIDANCE 3 (SAV3)/CYTOKININ INDUCED ROOT CURLING 1 (CKRC1), TRYPTOPHAN AMINOTRANSFERASE RELATED 1 (TAR1), and TAR2 (Stepanova et al., 2008; Tao et al., 2008; Yamada et al., 2009; Zhou et al., 2011). The biosynthesis of IAA from IPyA is catalyzed by YUC (YUCCA) flavin monooxygenase-like proteins (Mashiguchi et al., 2011; Stepanova et al., 2011; Won et al., 2011; Di et al., 2016b), which is a large family of 11 members that are functionally redundant with each other (Zhao et al., 2001; Cheng et al., 2006, 2007; Kim et al., 2007; Stepanova et al., 2011; Chen et al., 2014; Liu et al., 2017).

## The repressive histone mark H3K27me3 controls the expression of auxin biosynthetic genes

The DNA sequences in eukaryotes are assembled with proteins into nucleosomes, which are 146-bp of DNA wrapped 1.7 times around histone core protein complexes (H2A, H2B, H3, and H4). Surrounding the nucleosome is the junction histone H1, which compresses the nucleosome into higher-order structures to form chromatin fibers (Carlberg and Molnár, 2020b). In *Arabidopsis*, gene transcription is regulated by the chromatin state, which is determined by histone H3 methylation and acetylation and histone H2B monoubiquitination (H2Bub1) (Roudier et al., 2011). Compared with acetylation and monoubiquitination, methylation also occurs on lysine residues of histones but varies in the degree of modification, i.e., mono-, di-, or tri-methylation (Carlberg and Molnár, 2020a). Different methylation sites have different regulatory functions on gene transcription. For instance, H3K4me3 (H3 lysine 4 trimethylation) is detected specifically at active promoters, while H3K27me3 is correlated with gene repression over larger genomic regions (Carlberg and Molnár, 2020a).

About a decade ago, whole-genome occupancy studies showed that H3K27me3 was associated with the transcriptional repression of auxin-related genes. In comparisons of dividing and differentiated cells, differential H3K27me3 modifications were observed at the loci of *YUC*s, *Cytochrome P450*s (*CYP*s), and *TAA1*/*TAR*s (Lafos et al., 2011; He et al., 2012). In *Arabid*opsis, H3K27me3 is catalyzed by Polycomb Repressive Complex 2 (PRC2), which is an important epigenetic regulator of developmental processes (Schuettengruber et al., 2007; Muller and Verrijzer, 2009). In *Drosophila*, the PRC2 complex consists of four core subunits: the histone methyltransferase Enhancer of zeste [E(z)], Suppressor of zeste 12 [Su(z)12], Extra sex combs (Esc), and the histone-binding Nurf55 (nucleosome remodeling factor 55 kDa) (Schuettengruber and Cavalli, 2009; Simon and Kingston, 2013; Mozgova and Hennig, 2015). There are three PRC2 complexes in *Arabidopsis*, one of which is called FIS-PRC2 [including FERTILIZATION INDEPENDENT SEED2 (FIS2), Homolog of Su(z)12, CURLY LEAF (CLF), Homolog of E(z), FERTILIZATION INDEPENDENT ENDOSPERM (FIE), Homolog of Esc, and MULTIPLE SUPPRESSOR OF IRA 1 (MSI1), Homolog of Nurf55] (Mozgova and Hennig, 2015). During angiosperm fertilization, the auxin biosynthetic gene *YUC10* is constitutively repressed in maternal-derived tissues by the action of the FIS-PRC2 complex (Figueiredo et al., 2015). When FIS2, MEA/FIS, and MSI1 functions are deficient, the absence of H3K27me3 results in heterochronic expression of auxin biosynthetic genes, resulting in fertilization-independent development of empty seeds (Figueiredo et al., 2015).

**Figure 2:**
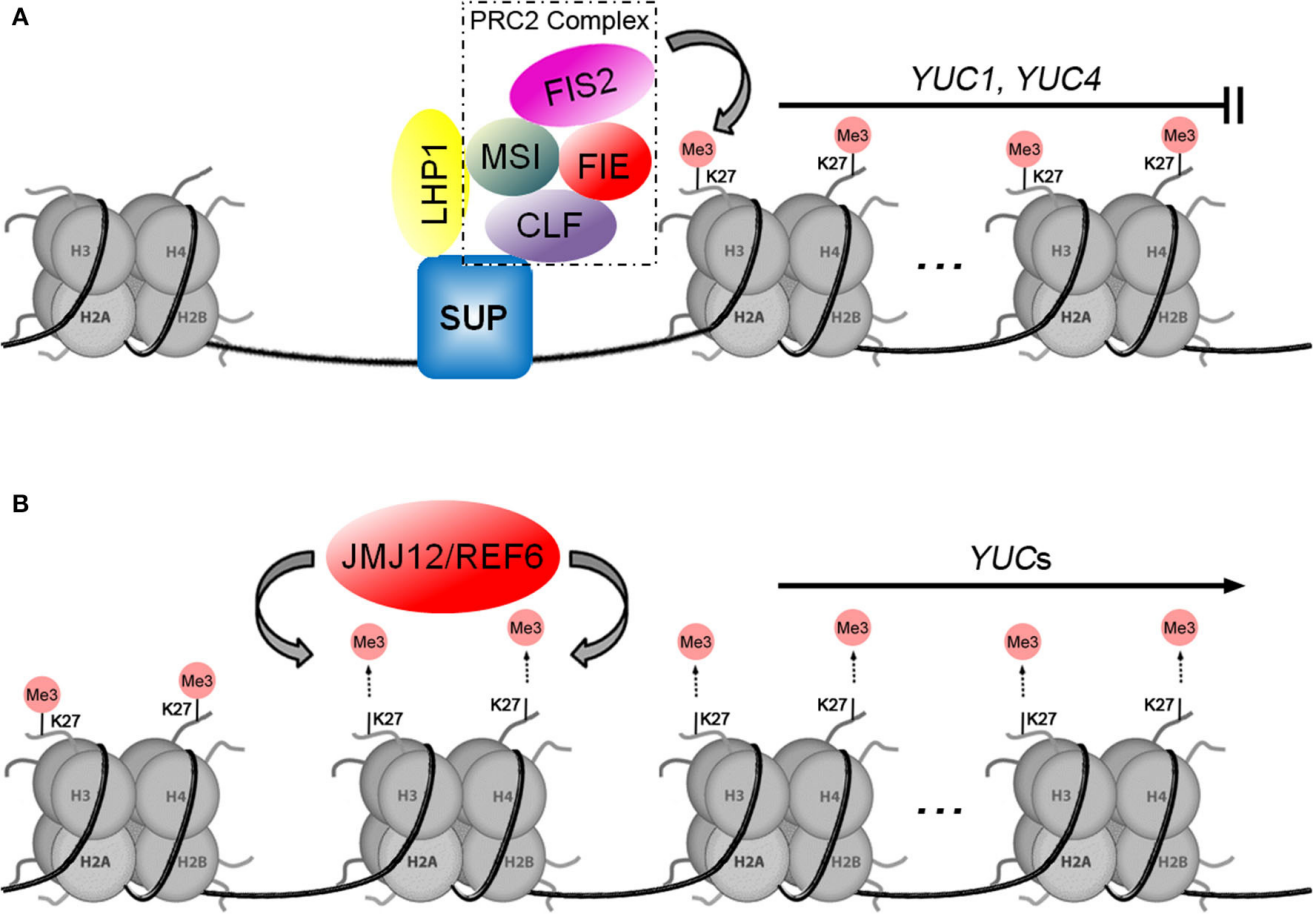
Two models for histone methylation are involved in the transcriptional regulation of *YUC*s. **(A)** Model of how H3K27me3 by the PRC2 complex inhibits the expression of auxin biosynthetic genes. **(B)** The JMJ12/REF6 histone demethylase positively regulates *YUC*s by demethylation of histone H3K27.

A model for the regulation of auxin biosynthetic genes by H3K27me3 was established through studies of LHP1/TFL2 (LIKE HETEROCHROMATIN PROTEIN 1/TERMINAL FLOWER 2), which is a unique homolog of *Drosophila* HETEROCHROMATIN PROTEIN 1 (HP1) in the *Arabidopsis* genome (James and Elgin, 1986; Gaudin et al., 2001; Kotake et al., 2003). It was initially thought that LHP1 binds to methylated H3K27, interact with a plant Ring-Finger protein, and catalyzes H2A ubiquitination as part of the plant PRC1-like complex (Xu and Shen, 2008; Bratzel et al., 2010). However, Derkacheva et al. (2013) found that the MSI1 subunit links PRC2 to LHP1 for H3K27me3. There is evidence of this LHP1-PRC2 association in other reports, such as LHP1 is proved to be co-purified with PRC2 and impacts H3K27me3 levels at thousands of loci (Wang et al., 2016), and the involvement of LHP1-PRC2 complex in maintaining H3K27me3 levels in dividing cells in *Arabidopsis* (Zhou et al., 2017). Early studies showed that TFL2/LHP1 is involved in auxin biosynthesis by activating the expression of *YUC*s (Rizzardi et al., 2011). Chromatin immunoprecipitation (ChIP) analysis showed TFL2/LHP1 enrichment over the transcribed regions of *YUC1, YUC2, YUC4, YUC5, YUC6, YUC8, YUC9*, and *YUC10* (Rizzardi et al., 2011). At the late stage of floral organ development, the C_2_H_2_-type zinc-finger transcription factor SUPERMAN (SUP) was expressed at the floral meristem-organ boundaries to define flower patterning. The loss function of SUP leads to higher levels of auxin in these boundaries, resulting in flowers with supernumerary stamens. As shown in Figure 2A, while binding to the *YUC1* and *YUC4* promoters through the C_2_H_2_ zinc-finger domain, SUP recruits the PCR2 complex by interacting with the CLF subunit and LHP1 to form the SUP-LHP1-PRC2 complex, which represses *YUC1* and *YUC4* expression by trimethylation modification of histone H3 at K27 (Xu et al., 2018). Interestingly, LHP1 functions by linking with PRC2 and participating in maintaining H3K27me3, which contradicts earlier studies that showed that LHP1 positively regulates auxin biosynthetic genes (Rizzardi et al., 2011), suggesting that the function of LHP1 seemed to be controlled by complex mechanisms. Perhaps LHP1 plays various biological functions with different epigenetic regulators in different tissues, at different developmental stages, or under different environmental stimuli. It remains necessary to uncover the precise mechanism by which LHP1 regulates auxin biosynthesis at the *YUC* loci in the future.

Gene repression by H3K27me3 is also orchestrated by other proteins that act antagonistically to PRC2. The JUMONJI DOMAIN-CONTAINING (JMJ) proteins have been identified to function as Histone DeMethylases (HDMs) in mammalian and plant cells (Agger et al., 2008; Lu et al., 2011; Cheng et al., 2018). As shown in Figure 2B, a number of studies have documented that JMJ12/REF6 (RELATIVE OF EARLY FLOWERING 6) was found to regulate *YUC1, YUC3, YUC7, YUC8, YUC9*, and *YUC11 via* specifically demethylating H3K27me3 and H3K27me2 in *Arabidopsis* (Cui et al., 2016; Li et al., 2016; Yan et al., 2018). In leaf explants upon wounding, upregulation of *YUC1* and *YUC4* is accompanied by the removal of H3K27me3 (Chen et al., 2016). Do et al. (2019) speculated in their review that the wound-jasmonate (JA) signal or even other environmental signals may trigger the recruitment of JMJ12/REF6. A recent study showed that JMJ14, JMJ15, and JMJ18 play key roles in high ambient temperature-induced *YUC8* expression. However, unlike JMJ12/REF6, the JMJ14, JMJ15, and JMJ18 mediated removal of H3K4me3 (activating) marks activates *YUC8* transcription by inhibiting the expression of some negative regulators (Cui et al., 2021).

Other studies have also suggested that H3K36me3 and H3K4me2 are involved in auxin biosynthesis. The H3K36me3 histone mark readers EMSY-Like protein 1 (EML1) and EML3 in the Tudor/Agent family repress *YUC10* expression during seed coat and endosperm development (Milutinovic et al., 2019). This is contrary to the common belief that H3K36me3 positively regulates gene transcription (Liu et al., 2010; Milutinovic et al., 2019), although the molecular mechanism remains unknown. The *RNA* binding protein FLOWERING CONTROL LOCUS A (FCA) is involved in chromatin silencing by promoting histone demethylation (Tian et al., 2019). A large number of studies have shown that PIF4 promotes growth by inducing the expression of *YUC8* at high ambient temperatures (Sun et al., 2012; van der Woude et al., 2019; Xue et al., 2021), but there is also a mechanism that decreases transcriptional activation of PIF4 to avoid heat stress damage. PIF4 recruits FCA to trigger histone H3K4me2 (activating mark) demethylation at the *YUC8* promoter region, which triggers PIF4 dissociation from the DNA to inhibit its expression (Lee et al., 2014).

## Histone acetylation and chromatin remodeling in activating or repressing auxin biosynthetic genes

Since histone acetylation was first reported (Allfrey et al., 1964), it has been extensively studied in microorganisms, animals, and plants (Anonymous, 2021). Histone acetylation neutralizes the positive charge of lysine at the histone tail, decreasing the interaction between the histone protein and the negatively charged DNA to promote an “open” and transcriptionally permissive chromatin structure (Lee and Workman, 2007; Lawrence et al., 2016). Increased levels of histone acetylation are associated with the activation of many genes involved in different plant biological processes, such as root morphogenesis (Nguyen et al., 2020), photosynthesis (Zhou et al., 2022), shade avoidance response (Peng et al., 2018), wound-induced cellular reprogramming (Rymen et al., 2019), and salt stress response (Li et al., 2014). The dynamic changes in histone acetylation are often controlled by two enzymes with opposing functions, HISTONE ACETYLTRANSFERASEs (HATs), and HISTONE DEACETYLASEs (HDAs) (McGinty and Tan, 2014). Current studies have elucidated that homeostasis of histone acetylation is not only critical for AUXIN/INDOLE-3-ACETIC ACID-AUXIN RESPONSE FACTOR (AUX/IAA-ARF) mediated auxin signaling (Nguyen et al., 2020), but also for regulation of auxin biosynthesis.

**Figure 3:**
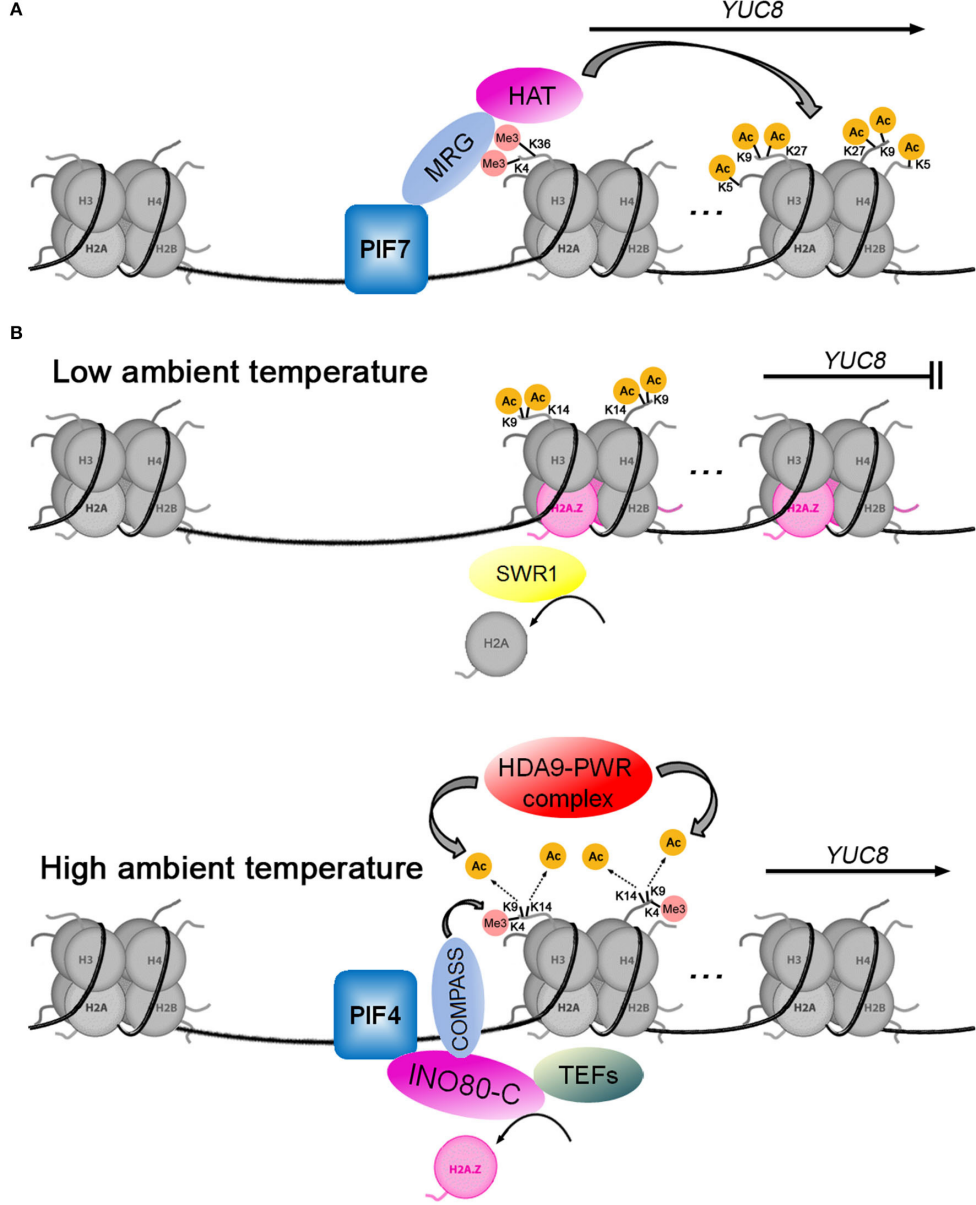
Two models for histone acetylation involvement in transcriptional regulation of *YUC*8. **(A)** The transcription factor PIF7 positively regulates *YUC8* by recruiting the MRG-HAT complex. **(B)** When the ambient temperature rises, the HDA9-PWR complex mediates the deacetylation of H3K9 and H3K14, which opens YUC8 chromatin to allow PIF4 binding to activate *YUC8* transcription. PIF4 recruits the INO80-C-COMPASS-TEFs complex to promote H2A.Z eviction, H3K4me3, and transcription elongation at the *YUC8* loci.

Local biosynthesis of auxin plays a key role in the shade avoidance response of plants. Disruption of auxin biosynthesis by mutation of the *TAA1* gene reduced hypocotyl elongation responses (Tao et al., 2008; Won et al., 2011). Shade can activate the expression of *YUC2, 5, 8*, and *9* (Tao et al., 2008; Hornitschek et al., 2012; Muller-Moule et al., 2016; Peng et al., 2018), which depend on different PIF transcription factors such as PIF4, PIF5, and PIF7 (Hornitschek et al., 2012; Peng et al., 2018). As shown in Figure 3A, Peng et al. (2018) have revealed the molecular mechanism of PIF7 activating *YUC8* transcription. When plants are exposed to shade, the PIF7 is dephosphorylated and subsequently binds to the G-box (CACGTG) *cis*-element of the *YUC8* promoter (Li et al., 2012; Peng et al., 2018). Secondly, PIF7 interacts with MRG2 (MORF-RELATED GENE 2), which binds to H3K4me3/H3K36me3 at the 5′-end of *YUC8* (Bu et al., 2014; Xu et al., 2014; Peng et al., 2018). Finally, the PIF7-MRG2 complex recruits HATs to promote histone acetylation at H4K5, H3K9, and H3K27 at the coding region of *YUC8*, resulting in the induction of its expression (Peng et al., 2018). A similar mechanism for transcriptional regulation of *GH3.3*, which converts IAA to IAA amino acid conjugates (Chen et al., 2010), is driven by the BASIC LEUCINE ZIPPER 11—TRANSCRIPTIONAL ADAPTOR 2b (bZIP11-ADA2b) complex, which is able to recruit GENERAL CONTROL NON-REPRESSIBLE 5/HISTONE ACETYLTRANSFERASE OF THE GNAT FAMILY 1 (GCN5/HAG1) to the *GH3.3* promoter, inducing the activation of *GH3.3* expression (Weiste and Droge-Laser, 2014).

As an evolutionarily conserved variant of the canonical histone H2A, H2A.Z plays a critical role in multiple cellular processes through its influence on chromatin structure and dynamics. In plants, H2A.Z is involved in regulating growth and development, phase transitions, and response to environmental stimuli (Kumar, 2018). One well-studied model of H2A.Z is the regulation of thermomorphogenesis through temperature-responsive nucleosome dynamics (Kumar and Wigge, 2010; Casal and Balasubramanian, 2019). The histone variant H2A.Z is enriched at gene loci with high thermo-responsiveness, and its presence is often associated with transcriptional repression (Coleman-Derr and Zilberman, 2012; Dai et al., 2017; Sura et al., 2017). Numerous auxin-related genes, such as *YUCs, AUX/IAAs*, and *SMALL AUXIN UP RNA* (*SAURs*), have been shown to mediate high-temperature-associated plant growth (Koini et al., 2009; Franklin et al., 2011; Sun et al., 2012; Lee et al., 2014; Perrella et al., 2022). As shown in Figure 3B, at low ambient temperature, the transcription of *YUC8* is repressed by the SWR1 chromatin remodeling complex (Tasset et al., 2018; van der Woude et al., 2019), which is necessary for inserting the alternative histone H2A.Z into nucleosomes in place of H2A (Krogan et al., 2003; Kobor et al., 2004; Mizuguchi et al., 2004). When the ambient temperature increases, the HDA9-PWR (POWERDRESS) complex mediates the deacetylation of H3K9 and H3K14 in the first nucleosome at the 5'-end of the *YUC8* gene, which reduces H2A.Z occupancy in nucleosomes (Tasset et al., 2018; van der Woude et al., 2019). The transcription factor PIF4 directly interacts with the INO80 chromatin remodeling complex (INO80-C), which mediates H2A.Z eviction at the *YUC8* loci (Xue et al., 2021). At the same time, INO80-C can interact with COMPASS (methyltransferase for H3K4 trimethylation) and TEFs (transcription elongation factors) to enhance H3K4me3 and transcription elongation (Xue et al., 2021). These dynamic changes in the chromatin promote *YUC8* expression, leading to plant morphological changes in response to elevated temperatures (Tasset et al., 2018; van der Woude et al., 2019; Xue et al., 2021).

There are two other reports which suggested that the chromosome remodeling resulting from histone deacetylation may also be involved in the transcriptional regulation of other *YUC*s. In the *Arabidopsis* gynoecium, the apical-basal and mediolateral polarity of auxin involve regulation at the *YUC4* loci through histone acetylation at H3K9 and H3K14 by GCN5/HAG1 (at −174 to −84 in the *YUC4* promoter, also the first nucleosome at the 5′-end of *YUC4*), which inhibits the transcription of *YUC4* (Poulios and Vlachonasios, 2018). The SWR1 complex consists of several subunits, such as PHOTOPERIOD INDEPENDENT EARLY FLOWERING 1 and ARP6 (ACTIN-RELATED PROTEIN 6) (Aslam et al., 2019). As a homologous protein of ARP6, ARP4 represses the transcription of *YUC9* through exchanging H2A with the histone variant H2A.Z (Lee and Seo, 2017), but it remains unknown if the release of *YUC9* transcriptional inhibition is related to HDA-PWR complex-mediated histone deacetylation. Moreover, in *Arabidopsis* leaf development, the expression of the auxin metabolism gene *IAMT1* (encoding an enzyme that converts IAA into its methyl ester) and biosynthetic gene *YUC6* were regulated by the subunit SWI3B of the chromatin remodeling complex SWI/SNF (Han et al., 2018; Lin et al., 2021).

Interestingly, the effects of histone acetylation on the transcription of *YUC8* were opposite in the two models (Figures 3A,B), the acetylation of histone activated *YUC8* transcription (Peng et al., 2018) during shade avoidance but inhibited *YUC8* transcription during thermomorphogenesis (van der Woude et al., 2019). We noticed that the nucleosomes containing acetylated histone were located in the coding region of *YUC8* in the study on shade avoidance, but were located at the 5′-end of *YUC8* in the study on thermomorphogenesis (Peng et al., 2018; van der Woude et al., 2019). In addition, the acetylated lysine sites were different, at H4K5, H3K9, and H3K27 during shade avoidance (van der Woude et al., 2019) and at H3K9 and H3K14 during high ambient temperature (Peng et al., 2018). This means that nucleosomes may have different functions based on the position of the acetylated histone in the chromatin and on which lysine residues in the histone are modified by acetylation. In addition, the histone acetyltransferase GCN5/HAG1 not only acetylates H3K27 in the *GH3.3* promoter to activate *GH3.3* expression (Weiste and Droge-Laser, 2014), but also acetylates H3K9 and H3K14 in the *YUC4* promoter to inhibit *YUC4* expression (Poulios and Vlachonasios, 2018). This further shows that compared with the enzyme that mediates histone acetylation modification, the position of acetylated lysine sites in histone H3 may determine whether transcription is activated or inhibited.

## H2Bub1 is involved in the regulation of auxin biosynthesis

Like histone acetylation, monoubiquitination of histone H2B (H2Bub1) is another important epigenetic modification related to gene transcriptional activation (Roudier et al., 2011). In nucleosomes embedded in the coding regions of genes with strong transcriptional activity, there are often increased levels of histone H2Bub1, which changes the chromatin state and is specifically linked with transcript elongation (Pavri et al., 2006; Bourbousse et al., 2012; Himanen et al., 2012; Feng and Shen, 2014; Van Lijsebettens and Grasser, 2014; Woloszynska et al., 2019). H2Bub1 participates in a variety of physiological processes by activating the transcription of related genes, such as seed germination and dormancy (Wang et al., 2022), flowering time (Cao et al., 2008; Woloszynska et al., 2019), defense (Zhao et al., 2020; Ma et al., 2021), and stress responses (Chen et al., 2019; Ma et al., 2019; Sun et al., 2020).

**Figure 4:**
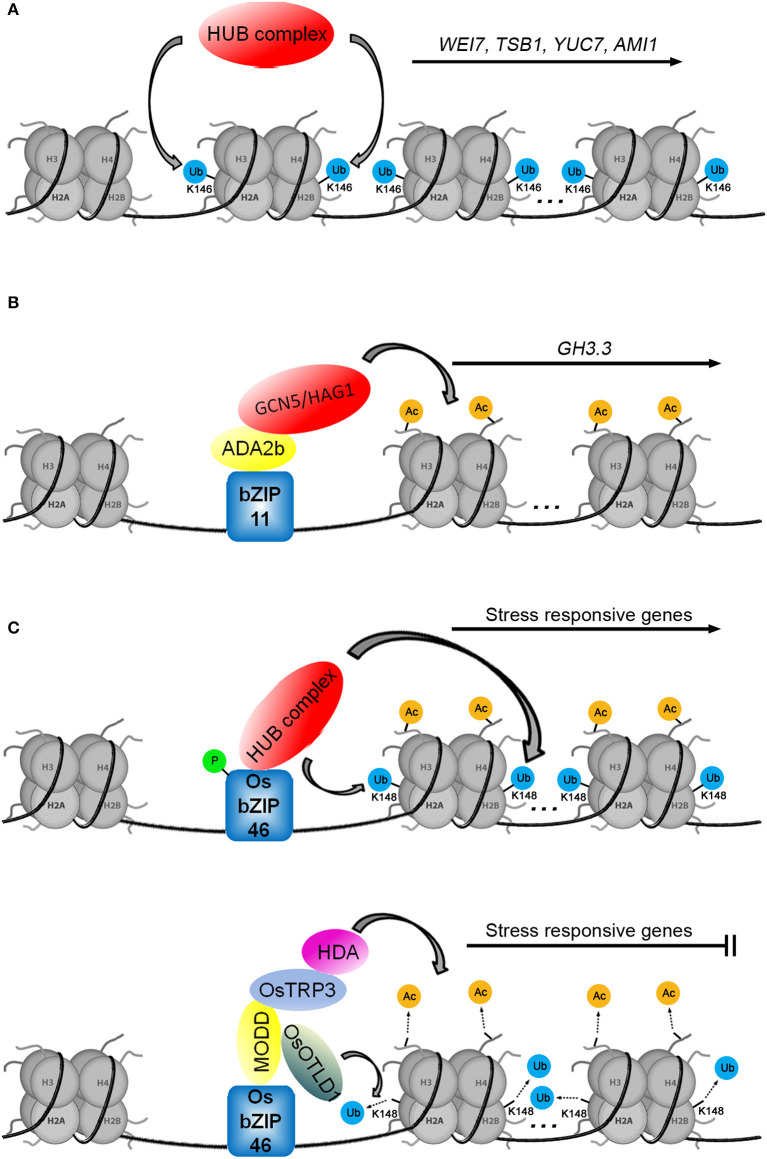
Three models for histone ubiquitination and/or acetylation are involved in transcriptional regulation. **(A)** The auxin biosynthetic genes *WEI7, TSB1, YUC7*, and *AMI1* are regulated by H2Bub1. **(B)** The transcription factor bZIP11 positively regulates *GH3.3* by recruiting the ADA2b-GCN5/HAG1 complex. **(C)** H2Bub1 (according to the study of Cao et al., 2008, we speculate that the monoubiquitination modification of H2B occurs at the K148 of H2B in Oryza sativa L.) and H3ac are involved in the regulation of stress-responsive genes (in Tang et al., 2016, the sites of histone lysine residues modified by acetylation were not mentioned).

The monoubiquitination of histone H2B is catalyzed by the ubiquitin ligase E3 heterotetrameric complex, consisting of two Histone Monoubiquitination 1 proteins (HUB1s) and two homologous HUB2s (Cao et al., 2008). The function of HUB1 was tied to auxin biosynthesis through the Cytokinin-Induced Root Curling (CKRC) system for screening auxin deficient mutants. There was a reduced level of IAA in the *ckrw2* mutant (*cytokinin-induced root waving 2*) that was linked to the loss of function of the gene *HUB1* (Wu et al., 2015). Recent ChIP analysis showed that the *ckrw1/hub1* mutant had corresponding defects in H2Bub1 in the coding regions of the auxin biosynthetic genes *TSB1, WEI7/ASB1, YUC7*, and *AMI*1 (*amidase 1*), indicating that H2Bub1 is required for the transcriptional regulation of these genes (Figure 4A; Zhang et al., 2021).

The current challenge in understanding HUB function is the identification of mechanisms that recruit the HUB complex to the auxin biosynthetic genes. Research on the rice transcription factor OsbZIP46 provides clues. Similar to how the transcription factor bZIP11 recruits the ADA2b-GCN5/HAG1 complex (Figure 4B; Weiste and Droge-Laser, 2014), phosphorylated OsbZIP46 recruits the HUB complex to activate the expression of stress-responsive genes by histone H2B monoubiquitination (Figure 4C; Ma et al., 2019). Working in opposition, the MEDIATOR OF OSBZIP46 DEACTIVATION AND DEGRADATION (OsbZIP46-MODD) complex, consisting of dephosphorylated OsbZIP46 and MODD, simultaneously recruits OTUBAIN-LIKE DEUBIQUITINASE 1 (OsOTLD1) and the TPL/TPR COREPRESSOR (OsTRP3)-HDA complex to shut down the transcription of stress-responsive genes *via* deubiquitination and deacetylation, respectively (Tang et al., 2016; Ma et al., 2019). The transcriptional activity of OsbZIP46 is blocked by the negative regulatory domain D. Phosphorylation promotes the interaction between OsbZIP46 and the HUB complex to activate the transcriptional activity of OsbZIP46 while also suppressing the negative regulatory function of domain D and preventing MODD interaction with domain D (Tang et al., 2016; Ma et al., 2019). The function of the OsbZIP46 transcription factor is involved not only in histone ubiquitination and deubiquitination but also in histone acetylation and deacetylation, suggesting that ubiquitination and acetylation may play a synergistic role in the regulation of gene transcription (Figure 4C). We speculate that some unknown factors may activate the transcription of auxin biosynthetic genes by recruiting the HUB complex to promote H2Bub1 on specific loci. Of course, there may be other epigenetic factors involved in this process.

## DNA methylation and small RNAs' role in auxin biosynthesis

Another epigenetic modification is DNA methylation which is usually associated with the transcriptional silencing of genes. DNA methylation is relatively stable but also reversible, and usually occurs on a cytosine base of DNA, to form 5-methylcytosine, in eukaryotes. Unlike mammalian methylation which occurs only in the CG sequence context, plant DNA can be methylated in CG, CHH, and CHG sequence contexts (with H representing A, T, or C) (Zhang et al., 2018; Gallego-Bartolome, 2020). There are two types of DNA methylation, one is *de novo* methylation, the methylation of DNA with two unmethylated chains; the other is maintenance methylation of a newly synthesized DNA strand after semi-conservative replication of methylated DNA. DNA methylation is mainly established by the *de novo* DNA methyltransferases DOMAINS REARRANGED METHYLTRANSFERASE 1/2 (DRM1/2), which are directed by 24-nt *small interfering RNA*s (*siRNA*s), and methylates all three sequence contexts *via* the RNA-directed DNA methylation (RdDM) pathway (Zhang et al., 2018). During DNA replication, methylation at the CG, CHH, and CHG contexts is maintained by METHYLTRANSFERASE 1 (MET1), CHROMOMETHYLASE 2 (CMT2), and CMT3, respectively (Stroud et al., 2014; Zhang et al., 2018).

**Figure 5:**
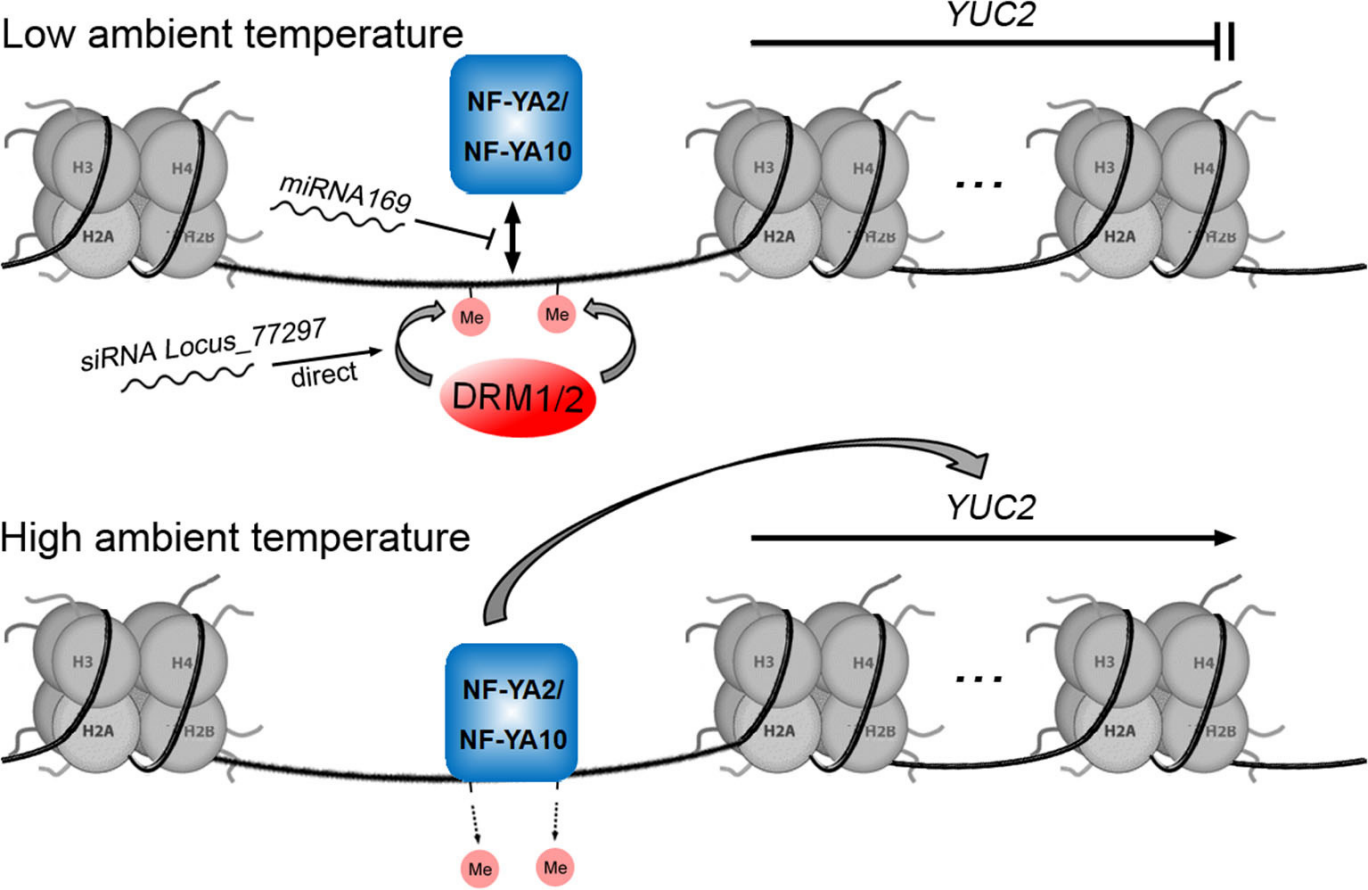
Model for the involvement of DNA methylation and small RNAs in the transcriptional regulation of the auxin biosynthetic gene *YUC2*.

In the previous section, we reviewed the regulation of auxin biosynthetic genes by PIFs and histone acetylation during thermomorphogenesis. Another study on thermomorphogenesis reported the temperature-related regulation of endogenous auxin biosynthesis by a PIF4-independent epigenetic pathway (Figure 5; Gyula et al., 2018). The expression of the auxin biosynthetic gene *YUC2* is negatively regulated by *miRNA169* which prevents the binding of transcription factors NF-YA2 and NF-YA10 to the *YUC2* promoter (Zhang et al., 2017). A 24-nt *siRNA, Locus_77297*, directs DNA methylation in the promoter of *YUC2*, which blocks the binding of NF-YA2 to inhibit the expression of *YUC2* (Gyula et al., 2018). At low ambient temperature, the high levels of *miRNA169* and *Locus_77297* result in a low concentration of active NF-YA2 and a methylated *YUC2* promoter. In contrast, at high ambient temperature, *miRNA169* levels decrease, as do *Locus_77297* levels and the methylation level at the *YUC2* promoter (Gyula et al., 2018).

It has also been reported that the auxin biosynthetic genes *YUC2* and *TAA1* are specifically up-regulated in leaves of the *drm1 drm2 cmt3* triple mutant, which has a low level of DNA methylation (Forgione et al., 2019). Surprisingly, the transcription of *YUC2* and *TAA1* showed almost no differences in the roots of the triple mutant, thus suggesting that DNA methylation is involved in tissue-specific patterns of gene expression (Forgione et al., 2019) and may be a potential mechanism to regulate local auxin biosynthesis. Recently, Markulin et al. (2021) found several gene loci targeted by RdDM in a whole-genome analysis. Most of the auxin biosynthesis-related genes (*TAA1, TAR1, TAR2, YUC1, YUC2, YUC5, YUC10, LEC2*) were targeted by RdDM. This means that DNA methylation is a very important yet poorly understood mechanism regulating auxin biosynthesis, marking methylation as an interesting subject that requires further study in plants.

## Concluding remarks

Auxin is an essential plant growth regulator that governs growth and development in concert with other signaling pathways. Therefore, understanding the auxin biosynthesis pathway and its regulation is crucial for plant science and agriculture. The biosynthesis pathway of auxin has two remarkable characteristics: the biosynthesis pathway includes multi-step reactions and has several parallel pathways (Figure 1). The complexity of auxin biosynthesis determines the complexity of its regulation. At present, there are only a few genes known to participate in the epigenetic regulation of auxin biosynthesis (Table 1), a simplicity that does not match the complexity of the pathway and suggests a lack of understanding of this topic. For example, little is known about the epigenetic regulation of the *TAA* gene family, which participates in the main auxin biosynthesis pathway. Moreover, multiple histone modifications act in a combinatorial fashion to specify distinct chromatin states (Carlberg and Molnár, 2020a). Therefore, future research should not only study the effect of certain histone modifications on the transcription of auxin biosynthetic genes but also study how various histone modifications determine chromatin structure and ultimately determine the specific expression level of genes. At the same time, the relationships between these histone modifications and DNA methylation, transcription factor binding, chromatin remodeling as well as RNA expression need to be further studied.

The three of the most critical questions for local auxin biosynthesis in plant growth and development are: in which tissues and at what time are the expression of auxin biosynthetic genes controlled by which internal and external signals and how these signaling pathways regulate chromosomal states. Although epigenetic regulation of gene expression during development has been known for decades, the specific relationship between auxin biosynthesis and epigenetic modifications in plants is only just being elucidated. The epigenetic regulation of auxin biosynthesis in plants is a fascinating subject requiring further study.

## Author contributions

LW drafted, wrote, and edited this review. J-LW helped LW to organize and collect information and participated in the writing of sections introduction, main pathway for IAA biosynthesis, and the drawing of figures. G-QG reviewed the manuscript before submitting it and gave many constructive comments. J-LW, D-WD, PL, LZ, X-FL, and LW participated in the discussion of this review. All authors have read and agreed to the published version of this manuscript.

## Funding

This research was supported by the National Natural Science Foundation of China (31970713), the Fundamental Research Funds for the Central Universities (lzujbky-2020-33 and lzujbky-2021-kb05), and the Natural Science Foundation of Gansu Province (20JR5RA270).

## Conflict of interest

The authors declare that the research was conducted in the absence of any commercial or financial relationships that could be construed as a potential conflict of interest.

## Publisher's note

All claims expressed in this article are solely those of the authors and do not necessarily represent those of their affiliated organizations, or those of the publisher, the editors and the reviewers. Any product that may be evaluated in this article, or claim that may be made by its manufacturer, is not guaranteed or endorsed by the publisher.
